# How to manage multiple food allergies in children

**DOI:** 10.1186/2045-7022-1-S1-S27

**Published:** 2011-08-12

**Authors:** Carina Venter

**Affiliations:** 1 University of Portsmouth, Portsmouth, UK

## 

Food hypersensitivity (FHS) is the umbrella term used for food allergies that involve the immune system and food intolerances that do not involve the immune system.

It is generally accepted that FHS affect about 6% of children and 3% to 4% of adults. It is less clear what the prevalence of multiple food allergies is, but allergies to two or three foods are often seen in highly atopic children. FHS has a huge impact on quality of life and any dietary advice given should aim to minimise this effect. This is a particular problem in children and adults suffering from multiple food allergies.

A clear diagnosis of the food allergies involved is very important as it well-known that patients often avoid more foods than indicated by a medical diagnosis. Despite many advances made in diagnosing and managing patients with FHS, the cornerstone of management still remains avoidance of the relevant food or foods. Patient will need information on food avoidance, understanding food labels and issues surrounding cross-contamination. In order to ensure that the diet is nutritionally sound, advice should be given about suitable food choices and following a healthy balanced diet including nutrient dense foods, whilst taking into account the dietary restrictions. Growth and development of children should be closely monitored and adult weight should be regularly assessed.

Practical issues that also need to be addressed include going on holiday, travelling and eating away from home, including school trips. The dietitian plays a crucial role in this process, but the need for a multidisciplinary team should never be underestimated. There are no standardised documents or protocols for the management of FHS and practices differ within and between countries.

Finally, if adrenaline auto-injectors are prescribed, correct administration should be demonstrated and reviewed on an ongoing basis and other atopic conditions should be closely monitored.

